# Why and how has the United Kingdom become a high producer of health inequalities research over the past 50 years? A realist explanatory case study

**DOI:** 10.1186/s12961-023-00968-w

**Published:** 2023-03-23

**Authors:** Lucinda Cash-Gibson, Eliana Martinez-Herrera, Joan Benach

**Affiliations:** 1 Department of Political and Social Sciences, Research Group on Health Inequalities, Environment, Employment Conditions Knowledge Network (GREDS-EMCONET), Universitat Pompeu Fabra, Mercè Rodoreda 24 Building, Campus Ciutadella UPF, Ramon Trias Fargas, 25-27, 08003 Barcelona, Catalonia Spain; 2 Johns Hopkins University-Pompeu Fabra University Public Policy Center (UPF-BSM), Barcelona, Catalonia Spain; 3 UPF Barcelona School of Management (UPF-BSM), Barcelona, Spain; 4 Research Group of Epidemiology, National School of Public Health “Héctor Abad Gómez”, University of Antioquia, Calle 62 No. 52–59 Bloque 33 Segundo Piso, Medellín, Colombia; 5 Ecological Humanities Research Group (GHECO), Universidad Autónoma, Madrid, Spain

**Keywords:** Health inequalities, Research capacities, United Kingdom, Critical realism, Mechanisms, Politics, History

## Abstract

**Background:**

Evidence on health inequalities has been growing over the past few decades, yet the capacity to produce research on health inequalities varies between countries worldwide and needs to be strengthened. More in-depth understanding of the sociohistorical, political and institutional processes that enable this type of research and related research capacity to be generated in different contexts is needed. A recent bibliometric analysis of the health inequalities research field found inequalities in the global production of this type of research. It also found the United Kingdom to be the second-highest global contributor to this research field after the United States. This study aims to understand why and how the United Kingdom, as an example of a “high producer” of health inequalities research, has been able to generate so much health inequalities research over the past five decades, and which main mechanisms might have been involved in generating this specific research capacity over time.

**Methods:**

We conducted a realist explanatory case study, which included 12 semi-structured interviews, to test six theoretical mechanisms that we proposed might have been involved in this process. Data from the interviews and grey and scientific literature were triangulated to inform our findings.

**Results:**

We found evidence to suggest that at least four of our proposed mechanisms have been activated by certain conditions and have contributed to the health inequalities research production process in the United Kingdom over the past 50 years. Limited evidence suggests that two new mechanisms might have potentially also been at play.

**Conclusions:**

Valuable learning can be established from this case study, which explores the United Kingdom’s experience in developing a strong national health inequalities research tradition, and the potential mechanisms involved in this process. More research is needed to explore additional facilitating and inhibiting mechanisms and other factors involved in this process in this context, as well as in other settings where less health inequalities research has been produced. This type of in-depth knowledge could be used to guide the development of new health inequalities research capacity-strengthening strategies and support the development of novel approaches and solutions aiming to tackle health inequalities.

## Background

Growing evidence demonstrates that avoidable and unfair systematic differences in health outcomes (i.e. health inequalities [HI]) [1] exist within and between countries [2–4]. Research on HI is essential to be able to assess the characteristics and trends of HI and to establish their causes, and can be used to inform the design and implementation of policy interventions aiming to reduce HI in different settings. A strong capacity to produce HI research at the local, national and global levels is therefore crucial to be able to understand and work towards addressing HI, yet this capacity does not exist worldwide [5, 6]. Despite notable advances and global efforts to invest in and strengthen such research capacities, further concerted efforts are still needed. In this paper, the term HI is used according to Whitehead and Dahlgren’s [1] conceptualization, and refers to all of the following terms: health disparities, HI, health inequities and social inequalities in health.

A recent bibliometric analysis of the global HI scientific production (1966–2015) identified significant inequalities within this research production worldwide [5]. The study also found the United Kingdom to be the second-highest global contributor to the HI research field after the United States [5]. Such findings raise important questions: why and how are some countries able to produce more research on this topic than others, and what types of mechanisms have been involved? Additionally, since scientific research output is generally considered as a proxy indication of research capacity, what do these diverse research outcomes suggest about HI research capacities in different settings? Furthermore, as an example of a “high HI researcher producer”, why and how has the United Kingdom been able to produce such a large volume of HI research during the last 50 years? Which key determinants and causal mechanisms might have been involved in generating this strong national HI research production and capacity over time?

In line with realist inquiries, our study therefore aims to generate valuable causal insights and knowledge about which mechanisms have worked in the United Kingdom, how, and under what conditions, for it to become a high HI research producer. Specifically, we aim to (i) understand why and how the United Kingdom has produced a high volume of HI research over the past five decades; (ii) test six theoretical mechanisms that we propose might have been involved in this national HI research production process over time and (iii) identify evidence to support, refute or refine these hypotheses.

## Methods

We conducted a realist explanatory case study, which included 12 semi-structured interviews with key informants. Data from the interviews were then triangulated with grey and scientific literature in order to strengthen our overall findings. Explanatory case studies attempt to explain causal relationships, and answer “how” and “why” questions. Realism is a strand of philosophy of science, and realist models of explanation attempt to consider the role of structure and human agency in social change. They aim to reveal the nature of hidden underlying causal forces (i.e. mechanisms) that are sensitive to different contextual conditions, and which can create series of changes that generate certain outcomes of interest [7–9].

We selected our “unique case of interest” (i.e. an example of a high producer of HI scientific research) based on previous findings of a recent bibliometric analysis [5]. Combining Pawson and Tilley’s [7] and Shankardass et al.’s [8] methodologies for realist evaluations and realist explanatory case studies, we developed our own realist explanatory case study protocol that explains how to design and implement realist explanatory case studies [9]. The study design included developing (i) a guiding abstract conceptual model based on existing literature on HI research production processes [10], (ii) a guiding context + mechanism = outcome (CMO) configuration and (iii) the rationale for proposing our theoretical mechanisms that we aim to test and refine through the case study [9]. The purpose of the guiding CMO configuration is to simplify the main process of interest (i.e. HI research production process) down to its key attributes [8]. This serves to “artificially isolate” the key combinations of factors that are embedded in specific historical, political and institutional contexts within the United Kingdom (C) and likely interacted over time to activate certain mechanisms (M), which collectively led to our outcome of interest (O) (i.e. high HI research production) [7].

Through our realist explanatory case study, we aimed to test six theoretical causal mechanisms (M1–M6) that we proposed based on a review of multidisciplinary literature. We hypothesized that these mechanisms might have been involved in this process, with the intention of refining them based on our study findings [7, 9]. Table 1 shows the CMO configuration that was created to guide our realist explanatory case study in the United Kingdom. (See the study protocol [9] for further details on how to design and implement realist explanatory case studies, and the rationale for proposing these six theoretical mechanisms.)

**Table 1:** CMO configuration used to guide our realist explanatory case study in the United Kingdom (adapted from [9])

Context (C1–4)	Mechanism (M1–6)	Outcome (O)
Structural (C1): Ideologies, government politics; HI exist in society; tradition of recognition of social and public health issues; minimum level of domestic resources to invest in health and social sciences Intermediary (C2): Institutional research funders; research institutions; stewardship Research infrastructure (C3): Minimum level of relevant human and information research capacities (e.g. available sociodemographic and health data; data collection systems; critical mass of trained professionals; scientific leadership; stewardship Research networks (C4): Scientific knowledge, financial and human resources	M1: Recognition with concern M2: Sense of moral responsibility to act M3: Stewardship for HI research M4: New resources to strengthen HI human resources M5: New resources to strengthen HI information resources M6: Cognitive social capital	O: High volume of HI research

Through the semi-structured interviews, we aimed to understand how and why the United Kingdom’s HI research field was initiated and how it has evolved over the past few decades. Study participants were initially identified from the published HI literature [5] and invited via email for interview if they met the following inclusion criteria: (i) senior researcher working or having worked in United Kingdom during the last five decades, of any gender; and (ii) has produced (and published) research on HI while working in United Kingdom during the last five decades. Out of the 13 people invited to interview, one potential participant declined to be interviewed.

Interview questions were developed using a political economy perspective and in line with our guiding abstract conceptual model, CMO configuration and supporting literature on HI research production and research capacities (refer to [9, 11] for details about the conceptual models). These research questions were tested in a pilot interview conducted by two of the authors and then adjusted accordingly to establish the core set of key questions for the rest of interviews. Participants were asked the following:

- Their professional background and initial motivation for working in the field of HI (to establish positionality)
- Why and how has the United Kingdom produced such a high volume of HI research over the past five decades, and why and how have certain institutions in the United Kingdom produced more HI research than others?
- What key historical, political, research and institutional events might have been important for the initiation and development of the HI research field in the United Kingdom over the past five decades, and why?
- Which factors have been important for developing national capacity (human and technical research infrastructure) to conduct HI research in the United Kingdom and why?
- Have individual or institutional ideologies and values been important for the process of generating our outcome of interest? If so, why?
- What role have research networks played in the HI research field over time?

Twelve interviews were conducted until saturation was attained [12]. In terms of the profiles of the study participants, the majority of the participants were male (*n* = 7) and professors (*n* = 11) who worked in different institutions and cities throughout the United Kingdom and had been trained in a range of disciplines, such as political and social sciences, medicine, public health and epidemiology, statistics and geography. Given the sample size, and the well-known profiles of many HI researchers from the United Kingdom, we do not provide further details to preserve study participant anonymity.

Participants signed an informed consent form prior to their interview, in line with ethics approval. Interviews were conducted in English by either one or two of the authors. Five interviews were conducted in person, and seven by teleconference. All interviews were audio-recorded, and one author was responsible for transcribing and translating the audio recordings, which were double-checked. All data were anonymized by the removal of any personal information that might reveal their personal identity. Participants were coded as P1–P12 in the results. The original and anonymized data (audio and transcripts) were stored separately in secure encrypted external hard drives that only the research team had access to. These data were iteratively triangulated with grey and scientific literature, which was identified through snowballing techniques and reviewed with the research questions and interview data in mind. One author initially coded the data using Microsoft Word 10 and analysed all the texts to identify recurrent themes, which were reviewed and agreed on by a second author [9]. Evidence from the various data sources was then synthesized, examined, interpreted and discussed between the authors until consensus was reached.

## Results

Through our case study, we found evidence to support our hypothesis that at least four of our proposed mechanisms (M) have been present and activated by a combination of contextual conditions (C) at different moments over the past five decades, which has likely led to the identified high production of HI research (O) in the United Kingdom [5]. In particular, we found strong evidence to support our hypotheses that M1 (*recognition with concern*) and M2 (*sense of moral responsibility*) have been present and activated during this process and time period and have contributed to the outcome of interest (O). In addition, based on our study findings, we refined several of our proposed mechanisms (M3, M4, M5 and M6) and identified two new potential mechanisms (M7 and M8) that might have been at play as well (Table 2).

**Table 2:** Causal mechanisms: proposed and refined during our realist explanatory case study

Proposed Mechanism	Refined mechanism	Comments
M1: Recognition with concern	M1: Recognition with concern	Findings suggest it acted as an M as well as a C in different moments to create O
M2: Sense of moral responsibility to act	M2: Sense of moral responsibility to act	Findings suggest it acted as an M to create O
M3: Stewardship for HI research	Stewardship and/or leadership for HI research	Findings suggest it acted as an M as well as a C in different moments to create O
M4: New resources to strengthen HI human resources	M4: Allocation of dedicated resources for HI research infrastructure	Refined to include the verb of action. Findings suggest it acted as the same type of M in both processes to create O
M5: New resources to strengthen HI information resources		
M6: Cognitive social capital	M6: Sense of cognitive social capital	Refined to include the verb associated with the theory. Findings suggest it acted as an M to create O
Potential new mechanism identified:	M7 (?): Misrecognition and/or denial	Limited evidence suggests this might have acted as an M to block the production of O or to activate other Ms to create O. To be tested and refined in future research
Potential new mechanism identified:	M8 (?): Identification of professional benefits (i.e. potential new intellectual territory) and/or scientific interests	Limited evidence suggests that this might have acted as an M to indirectly generate O. To be tested and refined in future research

### M1: recognition with concern

Strong evidence gathered from the different data sources suggests that M1 has been present in the United Kingdom during different moments over the past few decades, and has actively contributed to the initiation and development of the national production of HI research (O). Evidence also suggests that during different historical periods, “recognition” alone has acted as a contextual factor (C); however, once it is combined with “concern” it becomes activated, and together they act as a mechanism of change (M).

The United Kingdom’s production of HI research was established in the 1980s [3, 13–15], yet important questions are raised such as why and how was it established. Evidence suggests that “dramatic events” and/or perceptions of socioeconomic crisis [16–18] lead to public debate, recognition of and widespread concern about socially relevant issues (such as HI), which stimulates active investigation [14, 19, 20]. The following quotes illustrate this:

*I think it’s a kind of long running line of debate and concern, political concern…it was really about a kind of moral panic… there are these sort of moments I think, partly political, partly science based, and partly a kind of public outcry about social conditions.* (P11: Professor)

*You get a sudden collection of interests in social inequality, which may be because of either a change of government or a mini-revolution… and people may ask the question, why is there a lot of inequality in these country … So that’s the spark.* (P6: Professor)

Prior to the mid-1970s, there had been an economic crisis in the United Kingdom and an increase in social and HI [16, 17, 21, 22], which triggered “*public outcry* [with a] *growing public perception of a divided society*” [23] (p. 484). Also, after the establishment of the United Kingdom’s welfare state and National Health Service (NHS) in the early post-Second World War period, there had been a general assumption that population health would improve and HI would eventually decline, which they initially did [14, 15]. Yet, by the 1970s, they had increased once again, which raised concern over the effectiveness of the NHS and related public expenditure [15, 16, 21, 24, 25] (C).

Whitehead’s [10] *Action spectrum on inequalities in health* model includes recognition of HI as one of the initial activities (C). Whitehead explains that there is already a strong tradition of research and recognition of HI in the United Kingdom, dating back to the nineteenth century, when there were “*pioneering collectors of statistics, also offering social commentary on the data they gathered*” [10] (p. 480) (C). This, in combination with the new recognition of noticeable “*deteriorating socio-economic conditions [and] worsening health trends*” [10] (p. 472–3) during the 1960s and 1970s (C), and strategies of “*promoting awareness*” of the problems (C), raised “*voices of concern…about the extent of* [HI]” (C, and M1). Whitehead also mentions the role of “*professional advocates*” [23] (p. 487) and the “*intense professional pressures from health-related bodies and medical journals*” [23] (p. 483) (C), which in combination with various reports and other actions [26] helped to raise further awareness and interest in HI (C and/or M1). In addition, the author states that “*concern reached such a level by 1977 that the Labour government was persuaded to set up the* [HI] *Research Working Group, under the chairmanship of Sir Douglas Black*” [10] (p. 482) (M1).

The Black Committee was set up to assess national and international evidence on HI and draw up policy implications. The work of this committee led to the famous 1980 *Black Report* [13, 27]. The *Black Report* was said to have represented a significant shift in political thinking about HI [16]: it accumulated evidence that confirmed the existence of HI and showed the clear link between health and social position [15]. Evidence suggests that these findings sparked a key interest in HI and a growth in this research field, both in the United Kingdom (O) and abroad [3, 13, 27, 28].

We suspect that M1 might have also been activated in 1997, when HI were once again “recognized” as an important issue to be addressed (C) and were placed on the national political agenda by the New Labour (moderate social democratic party) government at the time [3, 15, 20, 29, 30] (C). This may have activated M1 at the political level, as the new government then commissioned an independent inquiry into HI, the so-called Acheson Report [31]. This report provided a comprehensive up-to-date synthesis of the HI scientific evidence and recommendations, mainly consistent with those of the *Black Report* [3, 15]. During this time, there was a strong political commitment to tackling HI [30], which in turn created favourable HI research conditions (C), such as an increase in dedicated HI research funding (C and/or M4) [15], resulting in more HI research being produced (O). This process may have occurred again in 2010, when the New Labour government commissioned the English review of the social determinants of health (SDH) (also known as the *Marmot Review*) to compile the latest evidence on HI [22, 32].

### Inhibition of M1: recognition with concern, or a potential new mechanism M7—misrecognition and denial

Interestingly, there is evidence to suggest that the lack of political recognition and concern (M1)—or even misrecognition and denial acting as a potential new mechanism (M7)—regarding HI during the 1980s and 1990s [10] was important to stimulate the generation of HI research (O). For the sake of chronological continuity in terms of the historical timelines of the HI research production process in the United Kingdom, we include this section on M7 here, since its contents will be important to understand the following sections.

By the time the *Black Report* was published in 1980 (despite having been commissioned by the former Labour government), the Conservative Thatcher government was in power, and evidence states that they were not keen to acknowledge the evidence and recommendations presented in the report [3, 20, 33]. However, the way in which the Conservative government released the *Black Report*, dismissed its findings and refuted the evidence on HI triggered an outcry by the public health community and top medical journals, as well as intrigue from the media [3, 18, 27, 33, 34] (potentially C and/or M1). As the following quote discusses:

*The publication of the Black Report in 1980 was absolutely pivotal… Its fame was fuelled by the fact that the government tried to bury it, and when it couldn’t, it tried to discredit it…that was like a red rag to a bull as far as the medical professional was concerned… and The Lancet and the BMJ… there was a feeling that it was being somehow pushed under the carpet, so as soon as journalists got wind of it, they thought “oh, there’s a story here, you know the government is trying to hide it”, so that helped circulate it.* (P8: Professor)

Findings suggest that the Conservative government’s negative reaction (C and/or M7) also “incentivized” certain individuals to act [3, 22]. Throughout the 1980s and 1990s, while the Conservative government was in power, there was a sociopolitical and scientific struggle for recognition of HI, both in and outside of academia, determined to prove that HI existed [9, 35] (M1 and M2), as the following quotes illustrate:

*Back in the ’80s, there was a real attack on any idea that health inequality was real, and a lot of us spent a lot of time on this… we had a big struggle to prove health inequalities exist.* (P4: Professor)

*As a result of Thatcher’s suppression of the health inequalities discourse… it sort of went underground, but equally true, it flourished outside the* [central] *government public sector…there were lots of Labour local authorities that produced what we used to call “local Black Reports”… and the third sector…* [all] *working together to keep the flag flying, and the concept alive.* (P1: Lecturer)

Furthermore, evidence suggests that the media and certain academic journals have been important for circulating the HI discourse over the years (C) [18, 28], due to their “*recognition of the importance of the issue*” [18] (p. 28) and willingness to publish material on the topic [3, 18, 26]. This likely helped to circulate or “diffuse” HI ideas (C), which were then picked up by others [10, 18, 36]. This process seems to have helped to circulate wider recognition and concern for HI (M1). The follow quote touches on this:

*When I first started doing research on health inequalities…* [people] *didn’t know whether they were higher at the top or bottom … then all the little bits of research on poverty and health, unemployment and health and so on… 10–15 years later you could talk to people … they’d ask what are you doing… you’d* [explain] *and they would say “what’s the point, isn’t it obvious?” and that was such a huge change. I think that was done though little bits and pieces, over time, by little bits of research coming out in the media* ... [creating] *a common sense that hadn’t existed earlier.* (P5: Professor)

### Evidence on M2: sense of moral responsibility to act

Strong evidence, particularly from the interviews, suggests that M2 has been present and has acted during different moments over the past few decades, which has contributed to the development of the national production of HI research (O). All participants reported that individual and institutional values, views and ideology have played an important role (C and/or via M2) in the HI research production process in the United Kingdom over time (O). The following quote explains this:

*Certainly, all of my research has been driven by my values…and my commitment—personally and politically—to social justice. So I don’t think my research is biased by that, but it’s driven by that…I think that it’s probably the case for anyone in this field. I just think that some people are more explicit about it than others… for me, health inequalities are profoundly political…You can depoliticize health inequalities in a research frame…but you can’t depoliticize the issue really.* (P2: Professor)

During the 1980s and 1990s, it was apparently difficult to obtain funding for HI research, and scholars have since reported that it was “*a lonely time*” for any HI researcher who decided to “*stick it out*” [37], and that their work was heavily scrutinized [28]. The presence of strong personal (egalitarian) values and a sense of moral responsibility to address social injustice seem to partly explain why some researchers remained so committed to working in this research field, despite the unfavourable working conditions (M2). In addition, several interviewees also stated that they thought that individual values and views, combined with different disciplinary perspectives and other factors (C), have been important to produce not only HI research (O), but also different types of HI research (i.e. focusing on more upstream or downstream determinants of health and HI). For example:

*There are researchers who would focus more on the psychosocial explanations, and there are researchers who would focus more on the social-material conditions, and would maybe have different values around that ... you get these very deep and personally felt controversies… I’m sure there is a whole mix… the psychological and the political, and the two are probably entwined.* (P11: Professor)

*Most people studying health inequalities…identify themselves as left-of-centre, but then there is a really big difference between how left-of-centre, and who they see as their allies…those kinds of personal relationships have an impact on how the field is shaped…there’s political and ideological, and kind of value-based things that everyone is bringing to the field, but they are also bringing their disciplinary training, and their personal likes…and all of those things interact.* (P3: Professor)

### Evidence on M3 potential refined: Stewardship and/or leadership for HI research

Findings suggest that stewardship and leadership existed at the individual and institutional levels during certain historical periods, which have helped to create an enabling HI research environment (C), and in turn lead to the production of HI research (O). It is unclear whether M3 should remain as “stewardship” or be refined to “leadership”, or whether these are potentially two different mechanisms. Some interview participants discussed the important role of individual HI scientific leadership; for example:

*Oh, it will be a story of individuals…a couple of plucky individuals who would have plugged away.* (P10: Professor)

*There have been some really key figureheads, who have set up institutions and they’ve attracted a lot of funding, got a strong reputation, and there’ve been people who have been training through them.* (P4: Professor)

Several participants also emphasized the importance of certain academic institutions as HI research stewards and leaders, due to their history and strong tradition within certain cities. These institutions have then attracted certain individuals to work in them (M3 at the individual level). As the following interviews illustrate:

*Some of it is the more disadvantaged cities…Liverpool* [for example] *… it’s very proud of the fact that the city council appointed the first medical officer of health in the country, and then the rest of the country followed, and he was very active in advocating for public health, so *[it] *has always felt that it’s had a tradition to uphold, and I think that Glasgow is the same*. (P8: Professor).

*I think that institutions or centres within institutions that have either unique or special access to key data are well positioned... For example, UCL* [University College London] * holds the ’46 and ’58, and ’70s, and also the millennium cohort study, so those are extraordinary resources...So I think there is a kind of science bit, but I also think that the politics of the city is really important…I guess it’s a combination of the history of the city, and the access to datasets…I’m sure lots of other people would say it’s the individuals. I think that maybe the individuals are drawn to cities that have a strong social history or politics. I am not a great believer that science is created by individuals, I think it emerges in particular contexts that are rich for certain sorts of research to develop.* (P11: Professor)

Evidence suggests that the United Kingdom’s national research funding institutions, such as the Medical Research Council (MRC), the Economic and Social Research Council (ESRC) [3, 15, 28, 37, 38] and the National Institute for Health Research (NIHR) in England [39–42], have played important roles in stewarding HI research (M3) at certain points over the past few decades, as well as in investing in and allocating resources to HI research infrastructure (see more examples under M4 on this point). However, evidence suggests that these national research funding institutions have mainly acted as HI research stewards (M3) within supportive political climates [18, 20, 21, 36, 37, 43]. The following quotes highlight this:

*A large chunk of the government funding comes through NIHR, through government sources, and that research agenda…so there is a kind of clear link between the political climate of the day and the type of research that gets funded.* (P2: Professor)

*Universities also respond en masse to where the funders are putting the money. So if they legitimize the studies, by doing calls and funding different groups, then the universities will recognize those groups and support them.* (P8: Professor)

### M4 refined: allocation of dedicated resources for HI research infrastructure—human resources

Findings suggest that the allocation of dedicated resources for HI research infrastructure, specifically related to human resources (M4 refined), has been activated by a number of contextual conditions during different periods, which has helped to produce HI research (O). Over the past five decades, there have been a range of MRC- and ESRC-funded initiatives (M3?) that have provided new resources for strengthening the HI research infrastructure in the United Kingdom, focusing on human resources (M4). These resources have potentially helped to produce HI research (O) via the activation of M3 amongst other things [3, 15, 38]. The following quotes explain the role of the ESRC in building national HI research capacities:

*ESRC…decide*[d] *to fund a big programme and they decided to do that shortly before we had a* [New] *Labour government…that made clear commitments to reducing health inequalities…and there were a lot of people who were trained during that programme*... [also] *there have been specific initiatives from the ESRC to train people in more kind of “more sophisticated quantitative” approaches at various points, and health inequalities researchers have kind of connected to that…* [which] *have been developed in quite a strategic, conscious way.* (P3: Professor)

*The ESRC got the health variations programme going…in terms of capacity-building it was very enormously successful…*[also] *the MRC set up a “health of the public” initiative which was pretty much the same…and then obviously the millennium birth cohort study was founded, and once you’ve got something like that, then you get a kind of gravitational pull of early career researchers who want to work on it for their PhDs… So I think it’s a combination of investment in research infrastructure, and then these grants which really provide stepping stones for early career researchers.* (P11: Professor)

In addition, the creation of new Master’s programmes, Doctoral fellowships and dedicated research groups within universities (M4 refined) have also been important to develop this particular HI-related human resource capacity, which has led to HI research (O). The following quote illustrates how this has might have occurred:

*There are a few senior figures, at various points they will have done some teaching, some PhD supervision…probably been involved in setting up courses, so then you get institutions settings up courses and programmes, specially focused on health inequalities…*[also] *people who are recognized for having expertise in an issue attract PhD funding and PhD students who want to study an issue.* (P3: Professor)

In addition, participants mentioned the 5-year Faculty of Public Health training programme in place that forms part of the NHS and has a strong focus on HI (M4 refined) [44]. As the following quote explains:

*The Public Health training programme in the UK *[is] *a large investment in a 5-year programme…it's traditionally been a kind of medical programme…but also because it’s been open to non-medics, it means that it is actually a much broader set of expertise…It’s very much framed around health inequalities…it generally takes that as its starting point, the historical work that has been done around health inequalities...*[and therefore] *there are basically advocates for that approach in every area across the whole country.* (P9: Professor)

### M4 refined: allocation of dedicated resources for HI research infrastructure—information resources

Evidence suggests that since the nineteenth century, there has been a strong recognition (and concern) of HI (C and M1) and of the need to have a strong research infrastructure in order to develop public health evidence and be able to inform policy and practice [15, 45, 46] (C). For example, in the nineteenth century, the Registrar General decennial censuses were developed in England and Wales, and the work of William Farr introduced the classification of causes of death [45, 46]. In the early twentieth century, THC Stevenson incorporated social class (e.g. occupation) into the official death statistics, which led to the Registrar General’s Social Class (RGSC) schema, and published a long series of reports on the distribution of HI by social class, which lasted into the early twenty-first century [22, 46–48] (C and/or M4 and potentially M1, M2 and M3). These resources laid important foundations from which a strong body of quantitative evidence on HI has been produced in the United Kingdom (O) [15]. The following quote explains why these have been important resources:

*Some of it is to do with the foresight of some of the people who set up some of the national data collection…the first census was* [in] *1838, and almost at the last moment they decided to add age and occupation to the death certificates, and that actually allowed you to do all sorts of things…so we were one of the few countries that actually had some measure of social class attached to the official births and marriages and deaths, so that was very fortuitous and farsighted of them to do that, but they were extraordinary social reforms as well...every 10 years the Registrar General does a social class analysis of all the data—the decennial supplements, and from the very beginning, they are powerfully written documents.* (P8: Professor)

Following that, the United Kingdom Office of National Statistics developed the Longitudinal Study that linked census longitudinal data to mortality [49], the British Regional Heart Study [18] and the Whitehall Studies, which were set up by various grants [15, 50] (M4). In addition, a range of MRC- and ESRC-funded initiatives have helped to strengthen technical resources for health and sociodemographic (e.g. HI-related) research through the country [3, 15, 38] (M4 and M3)*.* Furthermore, the famous British birth cohorts were created, apparently due to bottom-up foresight and pressure, and top-down investment [51] (M4). These cohorts have enabled more explanatory models of HI to be proposed (e.g. the psycho-social stress at work, social isolation and life-course perspectives/approaches) (O) [15, 27]. The following quotes illustrate the importance of these resources:

*I think there has been an enormous farsighted investment in datasets. Like some of the different birth cohorts that have been set up in the UK, fantastic longitudinal studies; that sort of infrastructures and resources and data that goes back a long way, and that requires investment over many decades to maintain it… and support for all sorts of people to use them… actually they are officially called the national treasures, so some people house the national treasures. So nurturing those is really important*. (P8: Professor)

*The birth cohort studies I think is the kind of unique bit of the UK infrastructure. It was all very accidental, and it was people working really hard to try and keep, get the money for the next wave…they got the ’46 money and then they said “right, we need to raise some money to go back”, so it’s been a wave by wave process... these things came together by good will and tenacity, so I don’t think the government had this overarching strategy, I think its scientists pushing very hard and Research Councils responding, and realizing that…I think it’s been very much a bottom up pressure...*[this] *creation of incredibly rich data*. (P11: Professor)

### M6 refined: sense of cognitive social capital

Evidence from different state sources suggests that the formation of informal and formal research networks has been extremely important to support HI researchers in a number of ways. These networks have acted as both platforms for and sources of new ideas through the sharing and exchange of knowledge and resources, and have contributed to the creation and activation of a strong sense of cognitive social capital (e.g. social values, norms related to social trust, solidarity/collective efficacy, sharing, and social participation and integration) (M6) [9]. This has likely interacted with other mechanisms (such as M1 and M2), leading to the coproduction of new HI research (O) [11, 34]. The following quotes illustrate this:

*It was probably 1976…I always remember thinking that we had a kind of telephone community, and because we all had shared an interest in social justice, we could ring each other up with questions and so on, and always know you would get some help…we were always of course reviewing each other’s research proposals and papers and so on.* (P5: Professor)

*Informal networks, I think that that’s really important…both within countries and between countries, it gives people the academic support, the intellectual support and the personal support…to continue to do the research over four decades, when over that period you get these massive shifts in the political climate.* (P2: Professor)

*The informal stuff is really important…I do think the formal networks kind of catalyse informal connections, connections of trust and respect, and it’s those that then become the kind of stepping stones to closer collaborations.* (P11: Professor)

In addition, the United Kingdom has a strong tradition of integrating different disciplinary perspectives and approaches to studying HI, which has been important for developing different types of HI research (O) and a better understanding of HI and their causes [11, 15, 36, 38, 52–54], as the following quotes explain:

*I think having a health inequalities research tradition that is open at its boundaries to social science has been incredibly important. So in the UK context, the overlap between social inequalities research and health inequalities research…and in the interest in class, and how inequalities and class are reproduced over generations. I think this has been probably an under-acknowledged resource for health inequalities research.* (P11: Professor)

*I guess a lot of the strength of UK research has come through people working… across disciplines and sharing expertise, and synthesizing their approaches… maybe that kind of public health tradition has helped to have a more interdisciplinary approach.* (P9: Professor)

These interactions seem to have been important for strengthening the national capacity to produce different types of research HI over time (O).

### Potential M8: identification of professional benefits (i.e. potential new intellectual territory) and/or scientific interests

Interestingly, we found some evidence to suggest that M1 and M2 may not have been the only mechanisms present and activated to stimulate or motivate certain academics to work in this research field over the past 50 years. Limited evidence suggests that the identification of other professional benefits (i.e. potential new intellectual territory) and/or scientific interests [18] (M8?) may have also been activated during certain historical periods, leading to HI research (O). The following quotes illustrate how the identification of scientific interests may have played a role in driving the production of HI research (O):

*I’ve always been left wing, and worried about social justice, but being more honest, the real reason I did so much work on* [it] *was just that the data was good.* (P10: Professor)

*My concern started with scientific interest, “gosh look at this, this is interesting, I wonder how we explain that”… it didn’t start with a strong political commitment... it’s not like my politics led me to do research on health inequalities…my political commitment grew stronger the longer I looked at the evidence, the more I did the research...when I was younger, I was just more interested in doing the research.* (P7: Professor)

The following quotes illustrate how the identification of other professional benefits (i.e. potential new intellectual territory) may have played a role in driving the production of HI research (O):

*Researchers who don’t have that feeling and passion, will just go where the money is… I mean now* [HI research] *is more fashionable than it was, better funded than it was, in this country anyway.* (P8: Professor)

*I think that there was a period in which health inequalities looked like a way of creating territory,* [an area] *that you could then get a job in.* (P4: Professor)

For example, with the presence and activation of M8, some researchers may have entered the HI research field at certain points, particularly when the topic became a political and research funding priority (C), and therefore “fashionable” to work on, and with more resources available to foster more HI research to be produced (O). This is in line with previous sociology of science research, which suggests that scientists view themselves as competing with one another for access to resources, credibility and intellectual territory (M8?) [18, 52, 53, 55, 56].

## Discussion

Our study provides important insights into why and how the United Kingdom has developed a strong capacity to produce a high volume of HI research over the past five decades. Using a realist explanatory case study approach, we were able to test our six theoretical causal mechanisms (M1–M6). Through the development of the case study, we found strong evidence to suggest that two of these mechanisms (M1 and M2), and potentially three others when refined (M3, M4 and M6 refined), have been present and activated, and have contributed to the United Kingdom’s HI research production process over the past five decades. This evidence suggests that the proposed M5 is potentially the same as M4, just with different types of HI research-related resources involved. We also identified limited evidence to suggest that two other potential mechanisms (M7 and M8) may have also been at play during different historical moments. Our findings also strongly suggest that there has been a combination of key historical socio–political–institutional contextual factors and conditions (C) that activated these mechanisms (M), leading to the increased production of HI research in the United Kingdom (O). Given the exploratory nature of this study, these mechanisms, factors and research processes warrant further investigation, both in the United Kingdom and in other global settings, particularly where lower volumes of HI research have been produced.

### How and why the United Kingdom’s HI research field was initiated and has developed

During the 1970s, there was some research produced on HI in the United Kingdom; however, evidence suggests that the national HI research field was not really established until after the publication of the *Black Report* in 1980 [3, 13–15]. The *Black Report* provided strong evidence on HI (O) and proposed different explanatory models for HI, which evidence states helped to guide further exploration of these ideas and generate more HI research (O) [13, 15, 22, 27]. In terms of the type of HI research produced, each disciplinary perspective frames the problem of HI differently and provides different accounts of their causes, which makes it challenging to reach consensus on which research findings to follow, and which political approach to take to try to address these inequalities [3, 11, 15, 16, 33, 52, 57]. However, over time, the United Kingdom has developed a tradition of integrating diverse disciplinary perspectives for the study of HI, which has created novel research and strengthened overall understanding about HI.

Whitehead [23] characterizes the diffusion of HI ideas during the United Kingdom’s Conservative political period of the 1980s and 1990s as “*confrontational*” [23], where “*political confrontation* [was] *met with denial”* [23] (p. 481) (M7?). It was mentioned during the witness seminar of the *Black Report* that “*the Government at the time was very sensitive to any criticism…they saw the inequalities issue as a direct attack on their policies, so they were very keen to counter any of those arguments”* [34] (p. 164). Scholars also mention how “*egalitarian ideas disappeared from public debate and those with a strong sense of justice became—in effect—closet egalitarians*” [19] (p. 298) (M7?). Availability of research funding to study HI was also extremely limited during that period [37], and there have been claims that the Conservative government attempted to suppress reports that presented evidence on HI, such as the *Black Report* in 1980 [34] and the *Health Divide* in 1987 [3, 34]*,* as well as official population data used to conduct research on HI [13, 18, 27, 58, 59].

Despite the hostile sociopolitical and research environment in the 1980s and 1990s (C), some HI research was still produced (O). Our case study findings suggest that the controversy around the *Black Report*´s publication, the lack of political recognition or concern about—or their denial of—HI (M7?) and the government’s hostility towards evidence of HI in fact fuelled some people to act (via M1 and M2) and produce evidence of HI [5, 10, 15, 25, 60]. Again, during the witness seminar of the *Black Report*, John Fox, a prominent HI research producer in the United Kingdom at the time, stated, “*The government’s disapproval motivated a lot of people…There was a lot of discussion about alternative explanations. And I think that promoted a lot of different research to help to address those issues*” [34] (p. 168). During this period, there appears to have been a persistent underlying “struggle for recognition” of HI [15, 25, 34, 35, 60], mixed with heightened concern (M1). In particular, a number of motivated individuals with strong egalitarian values and a strong sense of moral responsibility to act to address social injustices (M2) persevered in their research efforts (M3?) to prove that HI existed (O). Other research has also identified that many individuals working in HI research and policy in the United Kingdom “*frame health inequalities as a moral issue around which urgent action is required*” [61] (p. 82). In addition, Garthwaite et al. [52] identify different types of HI researchers within the United Kingdom who use distinct perspectives and approaches, which *“seem to relate to deeply held epistemological and ideological positions”* [52] (p. 475).

Interestingly, some limited findings also alluded to the potential presence and activation of M8?, whereby the identification of professional benefits such as access to resources, credibility and intellectual territory [18, 52, 53, 55, 56] and/or scientific interests, besides the activation of M2, may have motivated some individuals to enter this research field at certain points and therefore contributed to the HI research field (O). This mechanism may have been activated in particular when the topic became “fashionable” and a political and research funding priority (C)—for example, after 1997, when New Labour was in power and there was a strong shift in political focus towards addressing HI, although mainly towards the so-called downstream, individual-level determinants of health and HI [30] as well as a mandate for “policy-relevant” evidence to be produced (C) [30]. The National Research Council and other research funders subsequently placed HI as a priority research area (M3), which translated into the provision of more resources for strengthening HI research capacities (M4), fuelling the production of HI (O) [11, 41, 42, 62]. Whitehead (1998) has in fact characterized the *“diffusion of HI ideas*” during this time period as “*pragmatism*” [23] (p. 480). The availability of these new research funding pots and professional opportunities (C and/or M8?) might have incentivized other academics, with or without strong individual and egalitarian values (M2), to enter this research field and start to produce research on HI (O). Furthermore, it may have influenced the type of HI research produced.

Connected to this last point, substantial evidence emphasizes the key roles of politics (C) and research funding (C) in not only influencing the volume of HI research that is produced but also shaping (and framing) the type of HI research produced, and subsequently the type of HI research used to inform policy and practice [11, 15, 18, 36, 38, 52–54]. For example, after 1997, when New Labour was in power, there was a strong political preference towards promoting research focused on downstream determinants of HI [15, 30, 38]. This caused some scholars to raise concern over the predominant policy and research focus on “lifestyle drift” [52, 54, 63] and the “*limit[ed] opportunities to study the impacts of macro-level policy changes*” [52] (p. 473), which some study participants also touched upon. These findings therefore highlight the importance of evaluating the sociopolitical context in which HI research and research priorities are planned and implemented in different settings when evaluating HI research capacities [11].

Evidence shows how the United Kingdom has had a unique and strong tradition of recognition and foresight (C) for producing systematic, available and reliable health and sociodemographic data (M4 refined) that are used to produce evidence on HI [3, 15, 22]. The existence of such data has helped to greatly enhance overall understanding of HI [3, 15, 51]. Findings also identified a number of “stewards” and/or “leaders” of HI research, that is, committed individuals and groups and/or supportive institutions (M3?), which have helped to strengthen the national HI research infrastructure over time (M4 refined).

In addition, the formation of research networks (C?) has been important [18, 34], particularly informal networks at the beginning, which appear to have formed due to aligned perspectives (M1) and individual (egalitarian) values (M2) amongst other things. Such networks have helped to build trust, solidarity and a strong sense of cognitive social capital within the field of HI (M6). For example, during the *Black Report* witness seminar, it was mentioned that during the 1980s and 1990s, *“lots of people* [were] *supporting each other, strong networks* [were] *building up, which didn’t exist before that time”* [34] (p. 168). Interestingly, new public health funding initiatives, such as the MRC United Kingdom Prevention, Research, Partnership programme [64] (M3), recognize the importance of strengthening transdisciplinary research networks (C) in order to build and establish new perspectives to address complex issues (M6?), with a strong focus placed on the upstream determinants. The programme supports existing research networks, but also actively fosters the formation of new ones (C and/or M?). Hopefully, such initiatives will contribute to addressing concerns about past efforts being excessively focused downstream [52, 54, 63] and will lead to novel ideas and approaches on how to effectively tackle HI.

### Key learnings and recommendations

Important learnings can be derived from studying the United Kingdom’s experience of generating a high volume of HI research and in developing a strong HI research capacity in the process. We summarize these learnings in the form of recommendations:

- Widespread recognition and concern for HI amongst different agents (i.e. researchers, practitioners, policy-makers and civil servants, as well as civil society and the public) should be fostered. This includes recognition of the benefits of having solid, locally relevant evidence on HI, consistently produced over time, and used to inform future research, policy and practice. This should be accompanied by strengthening public awareness and literacy on HI and its potential causes, in line with the different disciplinary perspectives used to study HI.
- The formation of HI research networks should be prioritized and invested in, and should be formed across disciplines, sectors and institutions, to foster a sense of cognitive social capital and coproduce critical research and innovative solutions for tackling HI. In addition, HI-related communication and dissemination channels between different agents (i.e. researchers, practitioners, policy-makers and civil servants, as well as civil society and the public) should be fostered.
- Dedicated research funding for HI should be provided, which promotes the use of diverse, integrated, disciplinary perspectives and methods in HI research.
- Institutional and individual leaders and stewards for HI research should be fostered and invested in. In addition, investment in and allocation of HI-related human resources should be prioritized in order to foster scientific leadership in HI and develop a critical mass of HI-trained professionals at the local, regional, national and global levels.
- The investment and allocation of HI-related information resources should be prioritized to ensure that comprehensive, reliable sociodemographic and health data are consistently produced, collected, monitored and reported over time. This can then be used to inform future research, policy and practice.
- More HI research capacity assessments should be developed and conducted at the local, regional, national and global levels to identify HI research capacity strengths, weaknesses and potential information gaps. This valuable information can guide the development of more effective strategies to strengthen HI research capacities.

### Study strengths and limitations

As discussed in the study protocol [9], the realist, mechanism-focused approach can help to reveal previously hidden aspects of a process and an outcome of interest. This approach is highly relevant for answering our research questions and generating understanding on why and how a large production of HI research and strong HI research capacities have been created in the United Kingdom over time. To enhance the study’s rigour, and the validity and credibility of our findings, we provided prior justification for our case selection and proposed mechanisms, which were aligned with existing literature [5, 9]. We also triangulated different sources of data in order to test and refine these mechanisms [9, 65]. However, there are of course limitations to this approach. For example, since it attempts to simplify the process that leads to the outcome of interest (O), it reduces the predominant study focus to certain mechanisms (M). Therefore, the presence and interaction of a factor (C and/or M) is considered “relevant” only if it appears to cause a significant change to the outcome of interest (O); otherwise it is considered irrelevant and is “abstracted” away through the research process. As a result, other potentially important factors that might contribute to certain outcomes of interest may be missed.

Given the novelty of our work, we encourage more research that explores these mechanisms and processes further, both in the United Kingdom and in other global settings, as well as the perspectives and roles of other stakeholders. A similar historical in-depth realist explanatory case study was recently conducted in the city of Barcelona, which shares a number of similar findings [66]. A comprehensive comparison of these case study findings would be insightful; however, this is beyond the scope of this study. It will be particularly interesting to conduct similar case studies in contexts where a lower production of HI research has been found, to better understand why and how this outcome has occurred, and what some of the facilitating and inhibiting contextual conditions and mechanisms might be. Lastly, it should be noted that the focus of this study was the production of HI research, rather than the HI research usage process, which is considered to be a separate process [11] and has been studied in the United Kingdom context [36].

## Conclusion

Important learnings can be derived from the United Kingdom’s experience of generating a high volume of HI research over the past five decades and in developing a seemingly strong HI research capacity in the process. The case study takes a novel approach to exploring the HI research production process in the United Kingdom over the past five decades, and tries to identify the mechanisms and contextual conditions that are potentially involved in generating this high research output. We encourage more realist explanatory case studies to be conducted to explore the HI research production process in different global settings, particularly where less HI research has been produced. This type of in-depth knowledge can help to identify facilitating and inhibiting conditions and mechanisms, and could be used to guide future strategies for strengthening HI research capacities. Strengthening HI research capacities in different countries is essential for the ability to develop new locally relevant research ideas and evidence, which are needed to inform innovative action that aims to tackle HI and improve health for all.

## Data Availability

Anonymized data can be made available to the journal editors, if requested.

## Abbreviations

C: Contextual conditions
ESRC: Economic and Social Research Council
HI: Health inequalities
M: Mechanisms
MRC: Medical Research Council
NHS: National Health Service
O: Outcome of interest
SDH: Social determinants of health
UK: United Kingdom
